# p.P476S mutation of RBPJL inhibits the efficacy of anti‐PD‐1 therapy in oesophageal squamous cell carcinoma by blunting T‐cell responses

**DOI:** 10.1002/cti2.1172

**Published:** 2020-09-16

**Authors:** Lei Miao, Xiao‐Li Wei, Qi Zhao, JingJing Qi, Chao Ren, Qi‐Nian Wu, Da‐Liang Wei, Jia Liu, Feng‐Hua Wang, Rui‐Hua Xu

**Affiliations:** ^1^ State Key Laboratory of Oncology in South China Sun Yat‐sen University Cancer Center Collaborative Innovation Center for Cancer Medicine Guangzhou China; ^2^ Department of Pediatric Surgery Guangzhou Women and Children's Medical Center Guangzhou Medical University Guangzhou China; ^3^ Department of Medical Oncology Sun Yat‐sen University Cancer Center State Key Laboratory of Oncology in South China Collaborative Innovation Center for Cancer Medicine Guangzhou China; ^4^ Department of Pathology Sun Yat‐sen University Cancer Center State Key Laboratory of Oncology in South China Collaborative Innovation Center for Cancer Medicine Guangzhou China; ^5^ Precision Diagnosis and Treatment for Gastrointestinal Cancer Chinese Academy of Medical Sciences Guangzhou China

**Keywords:** ESCC, exceptional response, PD‐1, RBPJL (p.P476S), T cells

## Abstract

**Objectives:**

Anti‐PD‐1 immune checkpoint blockade represents the onset of a new era in cancer immunotherapy. However, robust predictors are necessary for screening patients with immune checkpoint‐responsive oesophageal squamous cell carcinoma (ESCC).

**Methods:**

We obtained biopsy samples from an ESCC patient with mixed responses. The expression of CD4, CD8, CD68, PD‐L1, RBPJL and IL‐16 was analysed by immunohistochemistry, and the correlation with prognostic value was obtained from the GEPIA portal. T‐cell functions were examined by flow cytometry, MTS and transwell assays. The secreted cytokines were identified using an Inflammation Array Kit. The concentration of soluble IFN‐γ was measured by enzyme‐linked immunosorbent assay. The clinical benefit of RBPJL was examined in a PBMC xenograft mouse model.

**Results:**

The patient had an exceptional clinical response with shrinkage of the primary oesophageal and lung metastatic lesions as well as enlargement of liver metastatic lesions after toripalimab monotherapy. Four liver‐specific gene mutations were identified. RBPJL showed better response to toripalimab in the PBMC cell‐derived xenograft (CDX) ESCC model. Conditional medium from RBPJL overexpression induced chemotaxis and proliferation of T lymphocytes, as well as Th2/Th1 differentiation through the RBPJL‐NF‐κB‐IL‐16 axis *in vitro*. These functions were all inhibited by the p.P476S mutation of RBPJL (RBPJL (p.P476S)).

**Conclusions:**

We report for the first time that RBPJL (p.P476S) promotes tumor growth in ESCC and inhibits the efficacy of anti‐PD‐1 therapy through blunting T‐cell responses. Our findings provide a potential new predictor for evaluating the efficacy of anti‐PD‐1 therapy in ESCC patients.

## Graphical abstract

We report for the first time that RBPJL (p.P476S) promotes tumor growth in oesophageal squamous cell carcinoma (ESCC) and inhibits the efficacy of anti‐PD‐1 therapy by blunting T‐cell responses. Our findings provide a potential new predictor for evaluating the efficacy of anti‐PD‐1 therapy in patients with ESCC.

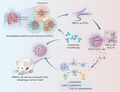

## Introduction

The current treatment options for oesophageal squamous cell carcinoma (ESCC), including surgery, radiotherapy and chemotherapy, moderately improve the outcomes of ESCC patients, including those with distant metastasis.^1^, ^2^, ^3^ With the approval of immune checkpoint inhibitors, programed death‐1 (PD‐1) checkpoint blockade for the treatment of patients with melanoma, lung cancer and other solid tumors has heralded the beginning of a new era of cancer immunotherapy.^4^, ^5^, ^6^, ^7^, ^8^ Different studies have shown that the expression of programmed death‐ligand 1 (PD‐L1) is highly associated with favorable overall survival, indicating that targeting PD‐L1 may be useful in PD‐L1‐positive ESCC patients.^9^, ^10^, ^11^, ^12^ Several studies have shown preliminary data on the safety and efficacy of PD‐1 inhibitors in treatment‐refractory ESCC.^13^, ^14^, ^15^ Generally, the safety profile is more acceptable and comparable to that in other tumors, but the objective response rate of PD‐1 inhibitors in ESCC is low, even in PD‐L1‐positive patients.^13^, ^14^, ^15^ Some studies have been designed to combine PD‐1 inhibitors with other drugs to improve efficacy, such as the RAMONA study.^16^ However, exploring the profound mechanism of failed immune response and the associated biomarkers is of vital importance.

Antitumor immune response is generated and activated in a subset of patients with various cancers, such as carcinomas of the head and neck, breast, lung and oesophagus.^17^ The recruitment and priming of tumor‐infiltrating T cells are highly responsible for the initiation and progression of cancer.^18^, ^19^, ^20^, ^21^, ^22^, ^23^ Moreover, some studies have demonstrated that the T‐cell‐inflamed phenotype correlates with positive treatment outcomes, which has been proposed as a prognostic biomarker in cancer. The findings of these studies suggest a correlation between clinical responses and immune cells in the tumor environment.^24^, ^25^ However, only a fraction of patients exhibit spontaneous T‐cell priming, which leads to a positive clinical response after exposure to PD‐1 checkpoint blockade agent. Robust predictors of treatment response in ESCC patients are currently lacking. Therefore, predictive biomarkers are needed during immune checkpoint therapy, which would be beneficial for selecting patients with immune checkpoint‐responsive ESCC.

Toripalimab^®^ (JS001) is a humanised PD‐1 IgG4 mAb developed in China and was the first anti‐PD‐1 antibody approved by the China Food and Drug Administration.^26^ The promising *in vitro* and *in vivo* efficacy of this drug has been reported.^27^, ^28^ The phase Ia study and phase Ib/II study, including an ESCC cohort treated with toripalimab monotherapy, were conducted at the Sun Yat‐sen University Cancer Centre.^29^ Herein, we conducted translational research on an ESCC patient with a mixed response to provide a potential predictor for evaluating the efficacy of anti‐PD‐1 therapy in ESCC patients.

## Results

### Assessment of biopsies and tumor response to toripalimab

**Figure 1:**
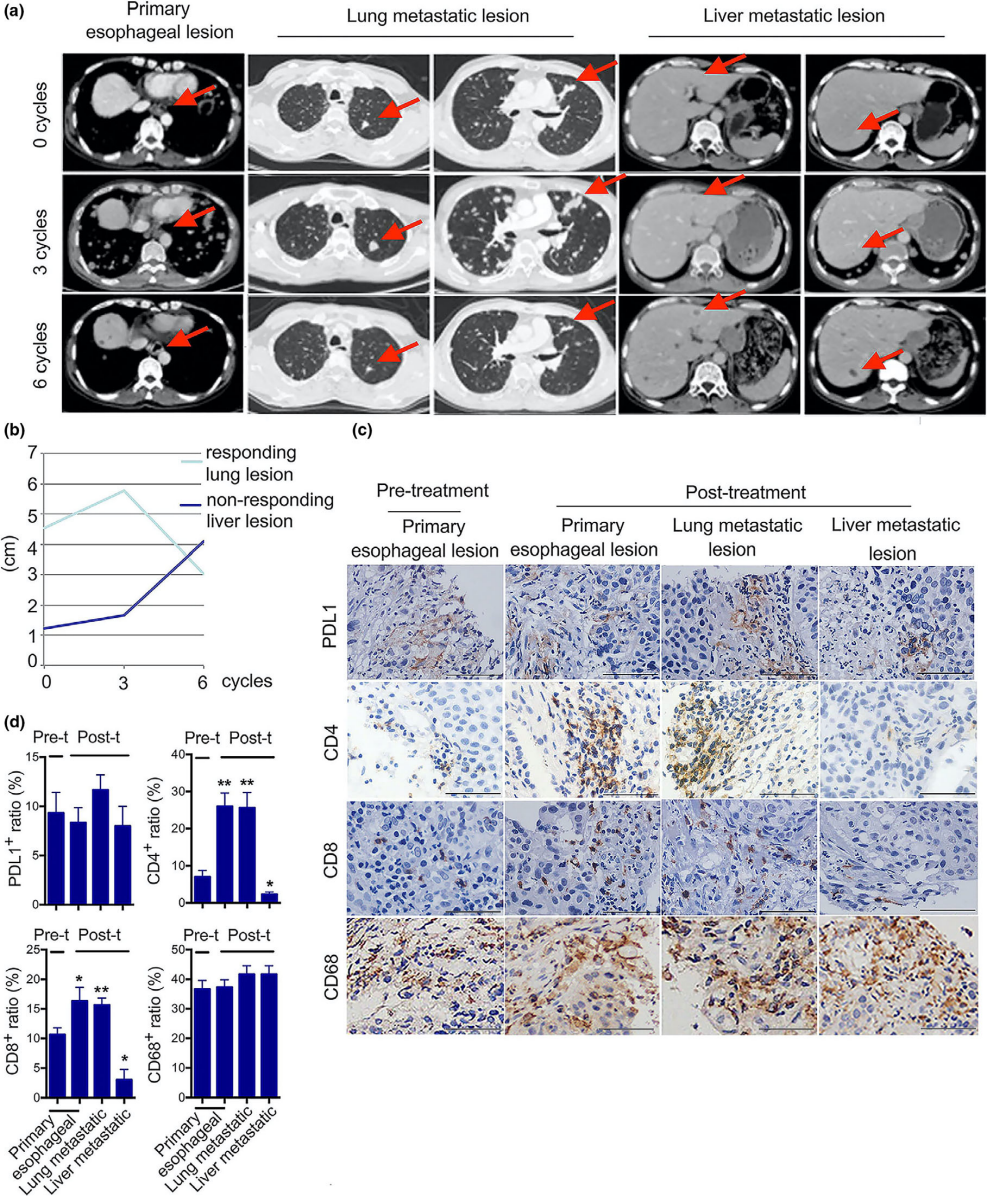
Tumor responses and immune profiling in primary tumor and metastatic biopsies exposed to toripalimab. **(a)** Computed tomography images of responsive lesions in the oesophagus and lung as well as non‐responsive lesions in the liver after 0, 3 and 6 cycles of toripalimab treatment. **(b)** Trends of the sums of the maximum diameters (cm) in lung and liver tumor lesions. **(c)** Primary and metastatic tumor biopsies of the patient at pre‐ and post‐treatment time points were obtained. Representative images of PD‐L1, CD4, CD8 and CD68 immunohistochemical staining in the tumor microenvironment. Scale bar, 200 μm. **(d)** The number of positive cells from 3‐5 fields was counted. The average positive ratio from each section was identified by symbol and colour. Data in **d** are presented as the mean ± standard deviation (*n* = 5). *P < 0.05, **P < 0.01. The data above are shown for three independent technical replicates.

In a male patient with ESCC, a mixed response to toripalimab was observed with shrinkage of the primary oesophageal tumor and most of the lung metastatic lesions, along with the enlargement of the liver metastatic lesions. The course of treatment in the patient is shown in Supplementary figure 1. Tumor responses of primary and metastatic lesions were assessed with a computed tomography scan every 6 weeks. Representative computed tomography sections are presented in Figure 1a. Although a CT scan, after three cycles (six infusions) of toripalimab, showed an increase in the size of the oesophageal and lung metastatic lesions (red arrows), a significant reduction in their size was observed after exposure to six cycles of toripalimab, which was most likely pseudoprogression. In contrast, we observed a continuous increase in the size of the liver lesions, which was considered insensitive to toripalimab. The sums of the maximum diameters of the representative tumor lesions are shown in Figure 1b. This mixed response encouraged us to investigate the in‐depth immune‐related mechanism that distinguished liver lesions from oesophageal and lung metastatic lesions.

### Immune profiling of biopsies from the primary tumor and metastases

Immune cell infiltration in the tumor microenvironment was first investigated with immunohistochemical (IHC) assay. The baseline biopsy samples from the patient showed pre‐existing remarkable expression of PD‐L1, low number of CD4^+^ and CD8^+^ T cells, and abundant CD68^+^ macrophages (Figure 1c and d). The biopsy samples obtained after toripalimab treatment showed significant intratumoral CD4^+^ and CD8^+^ T‐cell infiltration in primary and metastatic lung cancers but not in liver metastases, which theoretically supported our speculation of pseudoprogression. However, PD‐1 blockade treatment caused no significant differences in PD‐L1 expression and CD68^+^ macrophage infiltration at the tumor margins, compared with non‐treatment. Combined with computed tomography images, these results revealed that the patient was highly responsive to PD‐1 blockade with regard to primary and metastatic lung lesions but showed no response with regard to liver metastatic lesions, as reflected by the relative lack of CD4^+^ and CD8^+^ T‐cell infiltration in liver metastatic lesions.

### Genomic features of biopsies from the primary tumor and metastases

**Figure 2:**
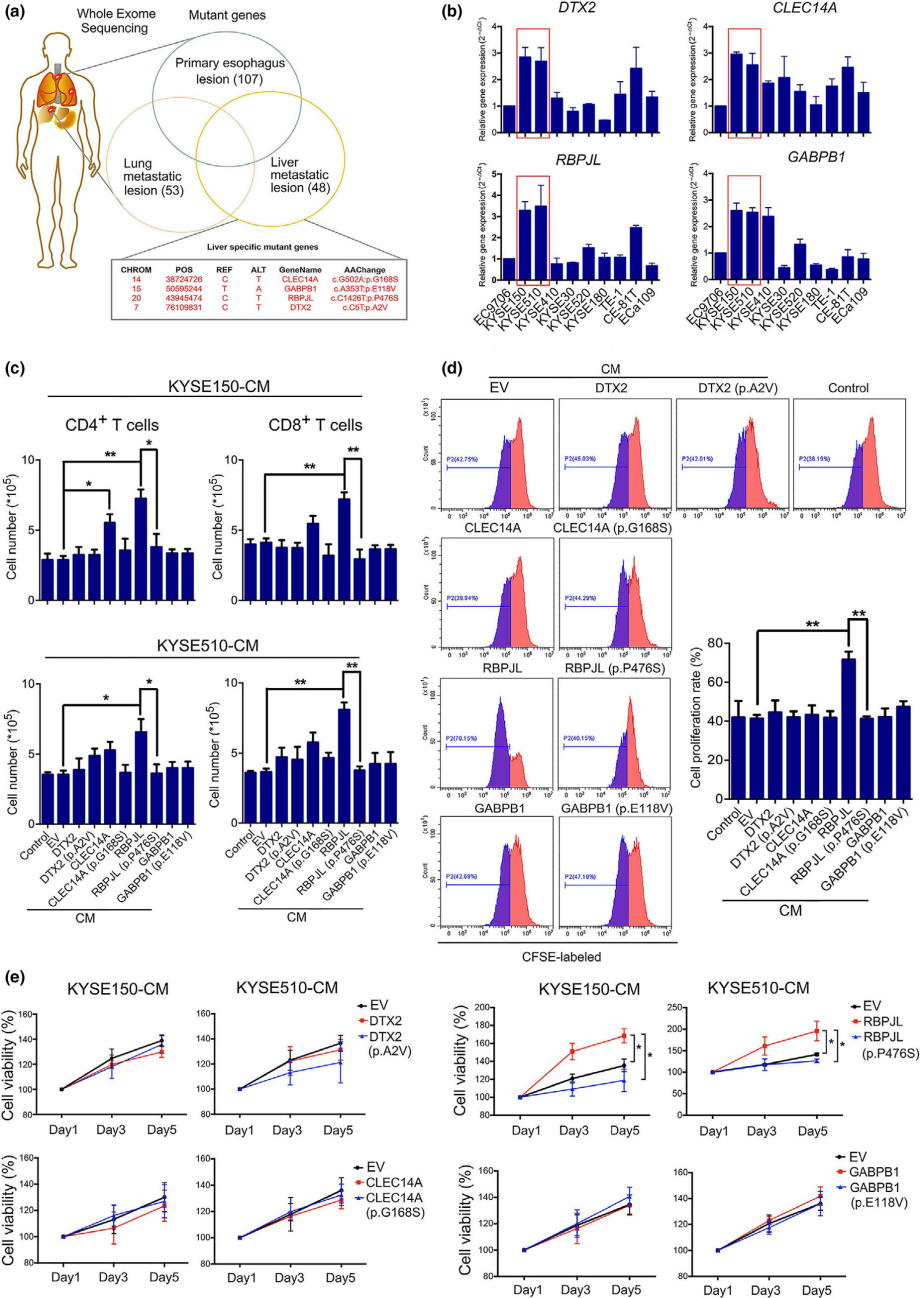
RBPJL (p.P476S) significantly decreased T‐cell chemotaxis and proliferation induced by CM from cells overexpressing RBPJL. **(a)** Four liver‐specific mutant genes after treatment were identified by whole‐exome sequencing, compared to primary oesophageal tumor and lung metastatic biopsies. **(b)** Reverse transcription‐polymerase chain reaction analysis of *DTX2*, *RBPJL*, *CLEC14A* and *GABPB1* expression in human ESCC cell lines. **(c)** The chemotaxis of CD4^+^ and CD8^+^ T cells was analysed by transwell assay. CMs from KYSE150 and KYSE510 cells overexpressing full‐length or mutant genes were placed in the bottom, and T cells were seeded in the top chambers of the transwell inserts. The number of T cells migrating to the bottom chamber was counted and analysed after incubation for 6 h. **(d)** Effect of different CMs from KYSE150 and KYSE510 cells on the proliferation of T cells was assessed by the loss of CFSE fluorescence after activation. Representative plots are shown. **(e)** The cell viabilities of different CMs incubated at various time points were determined by the MTS assay. Data in **c–e**are presented as mean ± standard deviation (*n* = 3), *P < 0.05, **P < 0.01. The data represent three independent technical replicates.

The pattern of responses in primary lesions and lung metastases as well as liver metastases led us to hypothesise that different responses to toripalimab in the patient were caused by immune cell‐mediated clonal selection and tumor outgrowth.^30^ To explore the genomic features associated with immunosuppressive response to toripalimab treatment, we extracted DNA from the biopsies of the post‐treatment primary and metastatic lesions, and subsequently performed whole‐exome sequencing. DNA extracted from whole blood was used as the germline control. All the tumor biopsies had modest overall specific mutational loads (107 nonsynonymous mutations in the primary oesophageal lesions, 53 nonsynonymous mutations in the lung metastatic lesions and 48 nonsynonymous mutations in the liver metastatic lesions) (Figure 2a). Moreover, by comparing the corresponding genes in primary and lung metastatic biopsies, we identified four liver‐specific nonsynonymous mutations in liver metastases, which have been reported to modulate immune cells. These mutations were located at *DTX2*, *CLEC14A*, *RBPJL* and *GABPB1* genes. Detailed information is shown in Figure 2a.

We first investigated the correlation between the RNA levels of the genes and survival outcome in ESCC patients from the TCGA database. Although we did not observe a statistically significant correlation with prognosis (Supplementary figure 2a), the top 15 % of *RBPJL* expression correlated better with moderate‐to‐better prognosis, than the bottom 15 % of *RBPJL* expression (the difference did not reach a statistically significant level). In addition, we assessed the other genes in TCGA database (Supplementary figure 3), and the expression of *CDH11X*, *TEX13A*, *LOR* and *GLRX2* showed good correlation with the survival outcomes of ESCC patients, but all the genes were barely reported as immune‐related functions. Therefore, we next focused on the functional effects of *DTX2*, *CLEC14A*, *RBPJL* and *GABPB1* genes and mutants, in order to clarify the further mechanism.

### RBPJL (p.P476S) significantly decreased chemotaxis and the proliferation of T cells compared with wild‐type RBPJL

Based on the data above, we speculated that the alteration of the genes in tumor cells might affect immune‐related genes or immune cell function. Moderate expression of the four genes was detected in the tumor lesions, and their expression in some ESCC cell lines was determined by reverse transcription‐polymerase chain reaction (RT‐PCR). Among the different cell lines, KYSE150 and KYSE510 demonstrated much higher expression of the four genes, with △Ct values ranging from 25 to 30, which were identified for further *in vitro* studies (Figure 2b).

To identify the effects of mutants, we constructed plasmids with full‐length cDNAs of *DTX2*, *RBPJL*, *CLEC14A* and *GABPB1* and the mutants DTX2 (p.A2V), RBPJL (p.P476S), CLEC14A (p.G168S) and GABPB1 (p.E118V), respectively. A significant increase in the expression of *DTX2*, *RBPJL*, *CLEC14A* and *GABPB1* after transfection was verified by RT‐PCR analysis (Supplementary figure 2b). Given that the mutations of these genes were identified from toripalimab treatment biopsies, we first analysed PD‐L1 expression upon overexpression of the four wild‐type and mutated genes, but we observed no quantitative differences, suggesting that PD‐L1 expression was not the main factor involved in the insensitive response of liver metastases in the patient (Supplementary figure 2d).

Furthermore, the effects of the mutants on T‐cell chemotaxis were further investigated. As shown in Figure 2c, conditional media (CM) obtained from the ESCC cell lines KYSE150 and KYSE510 overexpressing the four wild‐type or mutated genes and empty vector (EV) were placed at the bottom of the transwell inserts. Purified CD4^+^ or CD8^+^ T cells were seeded in the top chambers. Our data revealed that only CM of RBPJL significantly increased CD4^+^ and CD8^+^ T‐cell chemotaxis, compared with that of the EV group, which was reversed by CM from RBPJL (p.P476S)‐overexpressing cells. Other groups of CMs did not induce any changes. We also observed that the chemotaxis of CD14^+^ PBMC‐derived macrophages was not affected by any group of CM (Supplementary figure 2c), which was consistent with our IHC results.

T‐cell proliferation is an essential prerequisite for maintaining the stability of the T‐cell pool and chemotactic activity.^31^ Therefore, its proliferation under different CMs was evaluated. According to the MTS results, RBPJL markedly increased the proliferation of anti‐CD3/CD28‐activated purified CD3^+^ T cells in a time‐dependent manner, which was inhibited by CM from RBPJL (p.P476S)‐overexpressing cells. Moreover, CFSE‐staining assay also verified RBPJL‐induced proliferation after exposure to KYSE150‐ and KYSE510‐derived CMs for 5 days. However, for other genes/mutants, we failed to detect any differences among them (Figure 2d and e). Together, these data suggest that both the chemotaxis and proliferation of T cells were inhibited by the p.P476S mutation of RBPJL, which might be involved in the mechanism of insensitive treatment response.

### RBPJL (p.P476S) abolished Th1/Th2 differentiation induced by CM from RBPJL‐overexpressing cells

**Figure 3:**
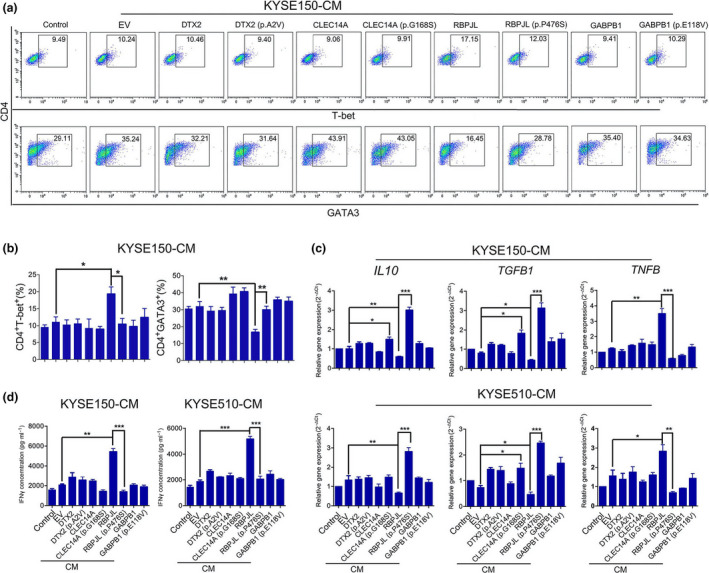
RBPJL (p.P476S) inhibited Th2/Th1 differentiation induced by RBPJL overexpression‐derived CM. **(a)** Sorted naïve CD4^+^ T cells were stimulated by anti‐CD3/CD28 and human IL‐2, and subsequently incubated in the presence of CMs from cells overexpressing the four full‐length and mutant genes for 5 days, followed by staining with CD4, T‐bet and GATA3 antibodies for flow cytometry analysis. **(b)** Percentages of CD4^+^ T‐bet^+^ and CD4^+^ GATA3^+^ T cells. **(c)** The levels of mRNA expression of *IL‐10, TGF‐B1* and *TNF‐B* mRNA in CD4^+^ T cells were determined by RT‐PCR analysis. **(d)** IFN‐γ concentration in CD4^+^ T cells was determined by ELISA. Data in **b–d** are presented as mean ± standard deviation (*n* = 3), *P < 0.05, **P < 0.01, ***P < 0.001. The data represent three independent technical replicates.

The effects of CMs from the cells overexpressing the four genes and mutants on CD4^+^ T‐cell differentiation were further investigated. As shown in Figure 3a–d, CM from RBPJL‐overexpressing cells significantly promoted Th1 differentiation as reflected by the increased numbers of CD4^+^ T‐bet^+^ T cells, upregulated the expression of *TNF‐β* and increased the concentration of IFN‐γ. However, Th2 differentiation was inhibited as reflected by the decreased numbers of CD4^+^GATA3^+^ T cells, along with the downregulated expression of *TGF‐β* and *IL‐10*. This conversion was also hindered by CM from cells overexpressing RBPJL (p.P476S), as reflected by the increased numbers of CD4^+^ GATA3^+^ T cells and decreased numbers of CD4^+^ GATA3^+^ T cells, along with increased expression of *TGF‐β* and *IL‐10* and decreased expression of *TNF‐β* and IFN‐γ. However, we failed to detect any differences among CMs from cells overexpressing *DTX2*, *RBPJL*, *CLEC14A*, *GABPB1* and the corresponding mutants, which indicate that Th1/Th2 differentiation was only influenced by the p.P476S mutation of RBPJL. We also examined the macrophage phenotype treated with different groups of CMs, but no obvious changes were observed (Supplementary figure 4).

### RBPJL (p.P476S) decreased the expression and secretion of IL‐16 induced by RBPJL

**Figure 4:**
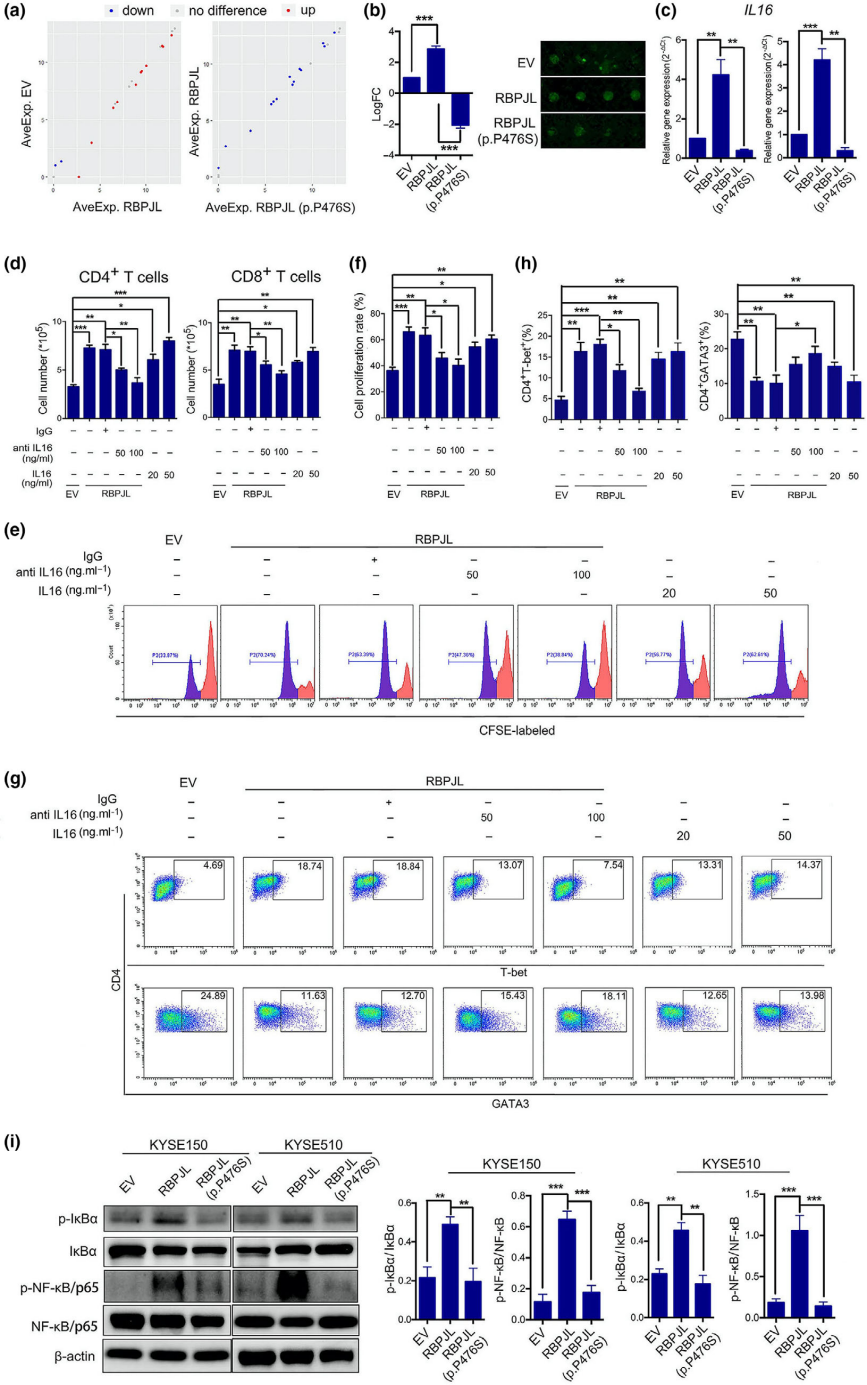
NF‐kB‐IL‐16 axis was involved in the chemotaxis and proliferation of T cells as well as Th1/Th2 differentiation induced by RBPJL overexpression. **(a)** Representative scatter plots compare the differences between EV and RBPJL and those between RBPJL and RBPJL (p.P476S) (red represents upregulation, blue represents downregulation, and grey represents no difference). **(b)** IL‐16 expression levels in CMs from EV, RBPJL and RBPJL (p.P476S) overexpression were measured and identified by quantitative measurement of 40 human cytokine arrays. **(c)** mRNA expression of *IL‐16* was determined by reverse transcription‐polymerase chain reaction. **(d)** Chemotaxis of CD4^+^ and CD8^+^ T cells as analysed by transwell assay. IgG was used as a control for anti‐IL‐16. **(e, f)** The effect of CMs from KYSE150 cells on the proliferation of T cells was assessed by the loss of CFSE fluorescence. **(g, h)** Sorted naïve CD4^+^ T cells were stimulated with anti‐CD3/CD28 and human IL‐2 and incubated in the presence of CMs for 5 days, followed by staining with CD4, T‐bet and GATA3 antibodies for flow cytometry analysis. Percentages of CD4^+^ T‐bet^+^ and CD4^+^ GATA3^+^ T cells are presented in **h**. **(i)** Immunoblot analysis of phospho‐IκBα, total IκBα, phospho‐NF‐κB/p65 and total NF‐κB/p65 in KYSE150 and KYSE510 treated as indicated, and β‐actin was used as a loading control. The ratio of p‐IκBα to IκBα and that of p‐NF‐κB to NF‐κB were quantitated. Data are presented as mean ± standard deviation (*n* = 3), *P < 0.05, **P < 0.01, ***P < 0.001. The data represent three independent technical replicates.

We have shown the effects of RBPJL and RBPJL (p.P476S) on the chemotaxis, proliferation and differentiation of T cells. To elucidate the associated mechanism, cytokines secreted by EV, RBPJL and mutant‐overexpressing KYSE150 cells were measured quantitatively with an array containing antibodies against 40 human cytokines. As shown in Figure 4a and b and Supplementary figure 5a, some factors associated with chemoattraction were altered under stimulation by different CMs. Among the cytokines, IL‐16 was the most remarkably elevated cytokine in CM from RBPJL‐overexpressing cells; however, the elevation was reversed by CM from RBPJL (p.P476S)‐overexpressing cells.

IL‐16 has been reported as a pleiotropic cytokine and serves as a chemoattractant for immune cells.^32^ However, the role of IL‐16 in cancer development and progression is still under investigation.^33^, ^34^, ^35^ To explore the effects of IL‐16 on T‐cell function in our study, we first validated the expression of IL‐16 in different groups of cells. As expected, mRNA expression of *IL‐16* was significantly elevated in RBPJL‐overexpressing cells compared to that in the EV group, whereas RBPJL (p.P476S) overexpression reversed the expression difference (Figure 4c). Furthermore, rIL‐16 dose‐dependently elevated chemotaxis, proliferation and differentiation of T cells. Subsequently, we depleted IL‐16 in CM using a neutralising antibody against IL‐16. Indeed, blocking IL‐16 significantly attenuated the chemotactic activity, proliferation and Th1/Th2 differentiation of T cells induced by CM from RBPJL‐overexpressing cells (Figure 4d–h).

### NF‐κB‐IL‐16 axis is involved in the chemotaxis and proliferation of T cells as well as in Th1/Th2 differentiation induced by CM from RBPJL‐overexpressing cells

**Figure 5:**
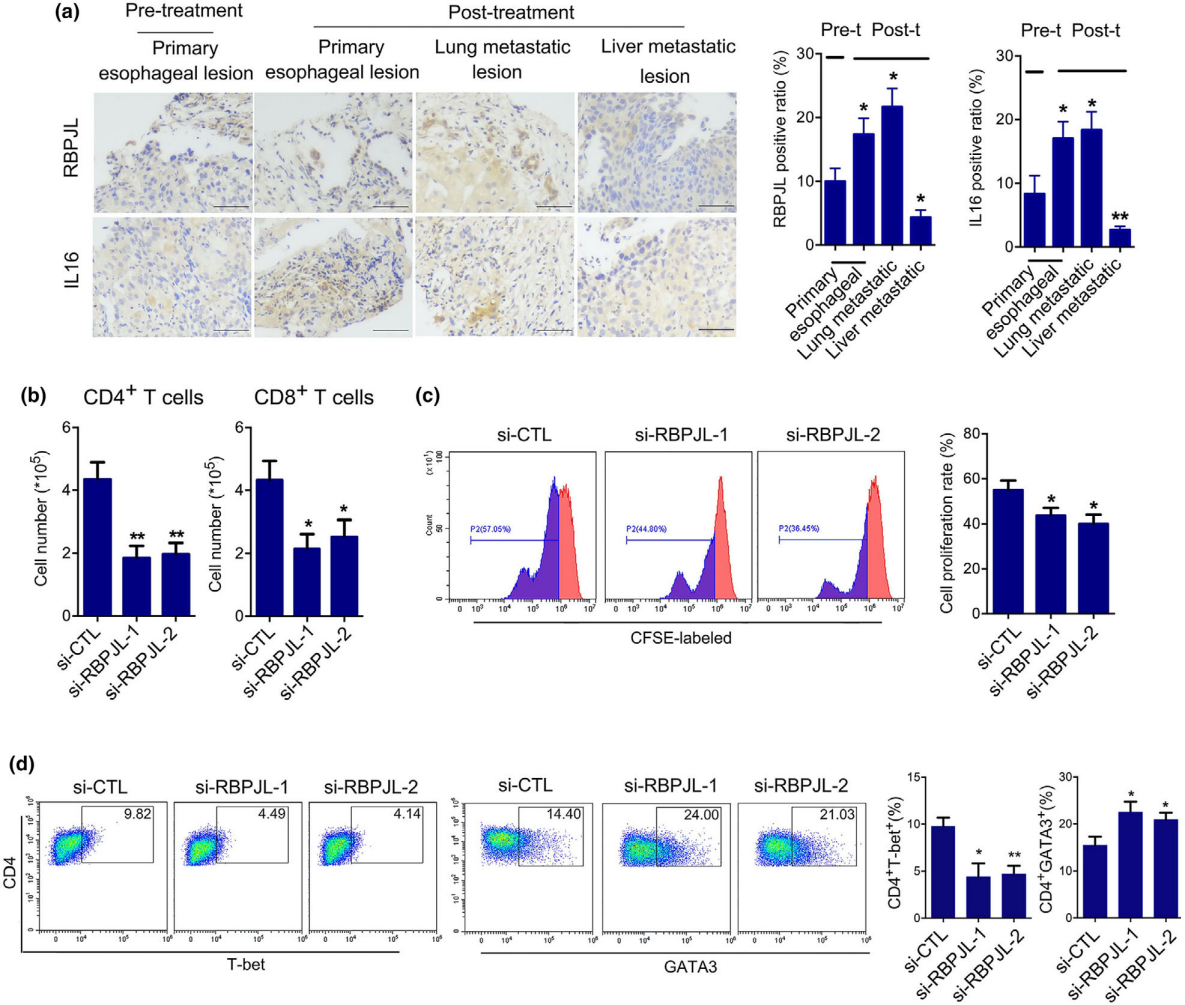
CM of RBPJL knockdown cells inhibited the chemotaxis and proliferation of T cells and Th1/Th2 differentiation induced by CM from RBPJL‐overexpressing cells. **(a)** Representative images of RBPJL and IL‐16 staining in responsive oesophagus and lung lesions as well as liver lesions. Scale bars represent 200 μm. The number of positive cells from 3 to 5 fields was counted. The average positive ratio from each section was identified. **(b)** The chemotaxis of CD4^+^ and CD8^+^ T cells as analysed by transwell assay. **(c)** Effects of CMs from KYSE150 cells on the proliferation of T cells were assessed by loss of CFSE fluorescence. **(d)** Sorted naïve CD4^+^ T cells were stimulated by anti‐CD3/CD28 and human IL‐2, and subsequently incubated in the presence of CMs for 5 days, followed by staining with CD4, T‐bet and GATA3 antibodies for flow cytometry analysis. Percentages of CD4^+^ T‐bet^+^ and CD4^+^ GATA3^+^ T cells are shown. Data are presented as the mean ± standard deviation (*n* = 3), *P < 0.05, **P < 0.01, ***P < 0.001. The data represent three independent technical replicates.

According to the quantitative analysis of 40 human cytokines in the CMs of different groups, functional annotation of differentially expressed cytokines in the Gene Ontology (GO) classification and Kyoto Encyclopedia of Genes and Genomes (KEGG) databases suggested that the TNF‐NF‐κB signalling pathway was closely associated with leucocyte migration induced by RBPJL and RBPJL (p.P476S) overexpression (Supplementary figure 5b). Therefore, we hypothesised that RBPJL might act upstream of NF‐κB to regulate the expression and secretion of IL‐16. Western blot analysis showed that RBPJL markedly increased the expression of phosphorylated IκBα and NF‐κB/p65 compared with EV control (Figure 5a), thus facilitating NF‐κB nuclear translocation in cancer cells. However, RBPJL (p.P476S) attenuated the nuclear translocation effects by decreasing the phosphorylation of IκBα and NF‐κB/p65. These data indicate that RBPJL (p.P476S) mutation suppresses the expression and secretion of IL‐16 by inhibiting the nuclear translocation of NF‐kB/p65.

### CM of RBPJL knockdown cells inhibited the chemotaxis and proliferation of T cells and Th1/Th2 differentiation

We further determined the expression of RBPJL and IL‐16 in the primary liver and lung metastatic lesions by IHC. Compared with the pretreated oesophageal lesions, the biopsy samples obtained after toripalimab treatment showed higher expression of RBPJL and IL‐16 in the primary and metastatic lung lesions, but lower expression in the liver metastases (Figure 5a). This led us to examine whether RBPJL (p.P476S) had a dominant‐negative effect on T cells. First, we generated siRNAs targeting human RBPJL and validated their knockdown efficiency, and as expected, RBPJL expression was reduced compared with that in the control (Supplementary figure 6a). Functional studies revealed that the chemotaxis and proliferation of T cells were decreased after treatment with CM from RBPJL knockdown cells. Moreover, RBPJL knockdown cell‐derived CM significantly promoted Th1 to Th2 differentiation, suggesting that RBPJL knockdown and RBPJL (p.P476S) had a similar effect on T‐cell function.

### RBPJL (p.P476S) reversed the effect of RBPJL on PBMC‐CDX oesophageal cancer model in the presence/absence of toripalimab

**Figure 6:**
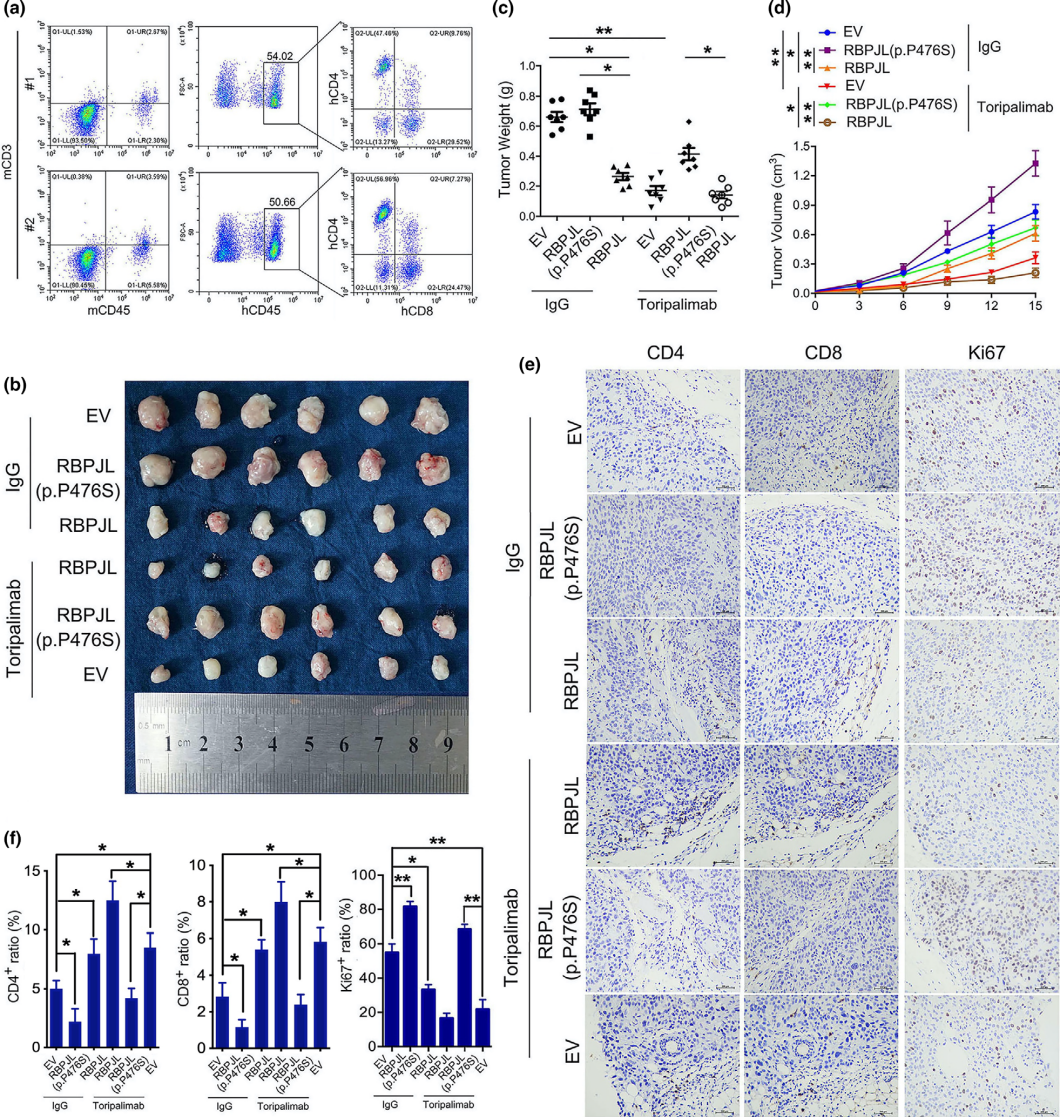
RBPJL (p.P476S) inhibited the therapeutic effects of RBPJL in a PBMCs‐CDX oesophagus cancer model in the presence/absence of toripalimab. **(a)** Representative flow cytometric plots of human CD45^+^CD3^+^CD8^+^, CD45^+^CD3^+^CD4^+^ T cells and mice CD45^+^CD3^+^ T cells in the blood of PBMC‐CDX NSG mice. Visual examination **(b)**, weight **(c)** and volume **(d)** of isolated tumors in mice subcutaneously injected with KYSE150 cells and treated as indicated. **(e)** Representative immunohistochemistry images of CD4, CD8 and Ki67 are shown in randomly selected tumors. Scale bar, 200 μm. The number of positive cells from 3–5 fields was counted. **(f)** The average positive ratio from each section was identified. Data in c‐f are presented as mean ± standard deviation (*n* = 6), *P < 0.05, **P < 0.01. The data represent three independent technical replicates.

The *in vitro* experiments showed the positive effect of RBPJL on T‐cell priming, and hence, we tested its functional activity in the antitumor effect of toripalimab *in vivo*. We established immune‐humanised PBMC‐CDX NSG mice and subsequently treated them as indicated. The rate of tumor formation with transplanted cancer cells was 100% for all the groups. The percentage of human CD45^+^ cells in the blood of PBMC‐CDX NSG mice was above 50% (Figure 6a); human CD4^+^ and CD8^+^ T cells are shown in Figure 6a. Compared with IgG, We found that toripalimab significantly inhibited tumor growth in humanised NSG mice. In addition, compared with the tumors in the RBPJL (p.P476S) group, RBPJL‐overexpressing tumors showed a better response to toripalimab, as reflected by the dramatic reduction in tumor weight and volume. Similarly, the RBPJL‐overexpressing group, not the RBPJL (p.P476S) group, exhibited significant tumor inhibitory effects in the absence of toripalimab.

Furthermore, we tested the reconstitution of T cells in PBMC‐CDX mice by using IHC staining. Compared with IgG, toripalimab significantly increased tumor‐infiltrating CD4^+^ and CD8^+^ T cells and inhibited tumor cell proliferation, as indicated by the downregulation of Ki67 expression, while the RBPJL‐overexpressing group showed more infiltration in the presence of toripalimab. In contrast, toripalimab had little effect on CD4^+^ and CD8^+^ T cells in the RBPJL (p.P476S)‐overexpressing group, while Ki67 was abundantly expressed (Figure 6e). Similarly, RBPJL‐overexpressing group exhibited greater infiltration of CD4^+^ and CD8^+^ T cells and tumor inhibition than the IgG group in the absence of toripalimab. There were no significant differences in CD4/CD8 ratio amongst groups (Supplementary figure 6b), in that the chemotaxis of CD4 and CD8 T cells was both inhibited by RBPJL (p.4476S) compared with wild‐type RBPJL. Taken together, these findings suggest that RBPJL (p.P476S) inhibits the antitumor effects of RBPJL *in vivo* in the presence or absence of toripalimab.

## Discussion

Given the exceptional clinical response of an ESCC patient with shrinkage of the primary oesophageal tumor and lung metastasis, and the enlargement of liver metastatic lesions after toripalimab monotherapy, we investigated and identified the p.P476S mutation of RBPJL as the genomic alteration responsible for the insensitive response of liver metastatic lesions to toripalimab therapy.

The clinical efficacy of PD‐1 checkpoint blockade is supported by the trafficking of tumor‐related antigen‐presenting cells and T cells.^36^ We first investigated the expression of PD‐L1, CD4, CD8 and CD68 in primary and metastatic lesions. According to the IHC results, there was a striking intratumoral CD4^+^ and CD8^+^ T‐cell infiltration restricted to the tumor margin in the primary and lung metastatic lesions, but not in the liver metastatic lesions. This is in accordance with the results of the computed tomography images, which showed pseudoprogression in the primary tumor and lung metastatic lesions as well as progression in the liver metastatic lesions.

Numerous genomic alterations, including nonsynonymous mutations that affect immune function, are associated with efficacy and improvements in objective response and long‐lasting clinical benefits, such as progression‐free survival.^24^ However, nonsynonymous genomic alterations can also initiate immune resistance, including profound local immune suppression, induction of tolerance and dysfunction in T‐cell signalling.^37^ To understand the genomic features and molecular mechanisms associated with the mixed responses of this patient, we obtained biopsy samples from the primary oesophageal tumor and the lung and liver metastatic lesions after exposure to toripalimab therapy, and the gene mutation profiles were compared by whole‐exome sequencing. Four liver‐specific nonsynonymous mutations, DTX2 (p.A2V), CLEC14A (p.G168S), GABPB1 (p.E118V) and RBPJL (p.P476S) were identified. Furthermore, the correlation between the RNA expression of the four genes and the survival outcome of ESCC patients was analysed. Unfortunately, only a high expression of RBPJL correlated with moderate‐to‐better prognosis (the difference did not reach a statistically significant level). This could be explained as follows: firstly, the data were obtained from the TCGA database, rather than PD1‐treated patients, and hence, the results did not reflect the impact of RBPJL expression on toripalimab sensitivity; secondly, RBPJL mutation might also be involved in secondary drug resistance, which requires further research in ESCC patients treated with PD‐1. The identification RBPJL mutation was intriguing, and we hypothesised that RBPJL (p.P476S) might not be sufficient to initiate ESCC predisposition but plays a role in tumor progression. Based on these findings, *ex vivo* studies on the functional effects of these genes and mutants were also conducted to clarify the further mechanism.

First, PD‐L1 expression in the pre‐ and post‐treatment tumor biopsies as well as in ESCC cell lines overexpressing the four wild‐type and mutated genes was examined. However, no noticeable quantitative differences were observed, indicating that PD‐L1 expression was not the only key factor associated with insensitive response to toripalimab therapy in the liver lesions of the patient. Given that most of the tumor‐related inflammation occurs as a result of the migration of leucocytes,^38^ the effects of CM from cells overexpressing the four wild‐type or mutated genes on T‐cell chemotaxis were examined. Intriguingly, only the RBPJL‐derived CM significantly increased CD4^+^ and CD8^+^ T‐cell chemotaxis, which was abrogated by the RBPJL (p.P476S)‐derived CM. Macrophage chemotaxis was also compared among these groups, but without positive results, which were consistent with our IHC results. Meanwhile, the proliferation of T cells exposed to CMs derived from different groups was also examined. We found that the CM from cells overexpressing RBPJL markedly increased T‐cell proliferation, which was blocked by the CM from cells overexpressing RBPJL (p.P476S). These results indicate that RBPJL‐derived CM might be a critical factor affecting the immuno‐modulatory capability of T cells.

Tumor‐infiltrating CD4^+^ T helper cells and CD8^+^ cytotoxic T cells are functionally compromised. CD4^+^ T cells are responsible for tumor initiation and progression,^39^ and naïve CD4^+^ T‐cell differentiation as well as balance of Th1/Th2 phenotype modulation is important in the modulation of tumor microenvironment.^40^ To check the activation state of T cells, the effects of CMs derived from different groups of cells on CD4^+^ T‐cell differentiation were further investigated. CM from cells overexpressing RBPJL significantly promoted Th2 to Th1 differentiation, and this conversion was inhibited by CM from cells overexpressing RBPJL (p.P476S). A potent immune response is regulated by the secretion of proinflammatory cytokines, such as IFN‐γ and TNF‐β,^39^ and hence, we determined the cytokines involved in Th1/Th2 differentiation. RBPJL (p.P476S) reversed RBPJL‐induced expression of *TNF‐β*, IFN‐γ and *IL‐10*. However, no obvious changes in the phenotype of macrophages were observed after treatment. The functional activities of RBPJL in the antitumor effect of toripalimab were further confirmed by *in vivo* studies. Human PBMCs were transplanted into tumor‐bearing NSG mice as a source of T cells. Consistent with *in vitro* experiments, RBPJL‐overexpressing tumors showed better response to toripalimab, which was reflected by the lower expression of Ki67 as well as the enhanced abundance and priming of T cells, whereas no significant response was observed in the RBPJL (p.P476S)‐overexpressing group. These data provide functional insights into the proinflammatory role of RBPJL and suggest that RBPJL (p.P476S) is particularly unfavorable to cancer patients with regard to toripalimab therapy.

Further studies on RBPJL are needed to fully delineate its mechanism of action. RBPJL is a canonical transcription factor that belongs to the Notch signalling pathway. The RBPJ/CSL effector transcription factor forms a complex with the membrane‐tethered intracellular domain of Notch receptors (Notch1‐4) in the nucleus, resulting in the activation of transcription of the target genes.^41^ Many RBPJ‐related target genes are also targets of NF‐κB (nuclear factor kappa B, http://bioinfo.lifl.fr/NF‐KB/). As one of these transcription factors, the predominant form p65/p50 of NF‐κB exists in the cytoplasm and is maintained in an inactive state through its interaction with IκBα. When exposed to stimuli, IκBα is phosphorylated, released and translocated to the nucleus to activate the transcription of genes.^42^

Functional annotation of altered cytokines in GO and KEGG databases showed that the NF‐κB pathway is closely associated with leucocyte migration induced by *RBPJL* and RBPJL (P476S) overexpression. This was consistent with the report of previous studies, which stated that Notch‐RBPJ signalling promotes disease progression through a process largely driven by NF‐κB‐mediated inflammation.^41^ Western blot analysis also showed that, compared with the EV control, RBPJL markedly increased the expression of phosphorylated IκBa and NF‐κB/p65, which was blunted by RBPJL (P476S). NF‐κB is a master regulator of inflammatory responses, both in the initial stages of inflammation and in the resolution phases.^43^ The interplay between Notch and NF‐κB has been described to produce an adequate inflammatory response, and leads to either activation or repression of gene transcription.^44^ Furthermore, the synergism between Notch and NF‐κB in macrophages is essential for interleukin expression^45^, ^46^ and macrophage activation,^47^ which has been demonstrated to facilitate protein shuttling and retention in the nucleus through protein–protein interactions.^48^

Cytokines, which are key factors in leucocyte migration and chemotaxis, can shape the immune and biological phenotypes of tumors as well as regulate the proliferation, invasiveness and metastasis of tumor cells.^49^ Interleukins are implicated in inflammatory responses^50^; as measured by quantitative human cytokine arrays, we showed that *RBPJL* overexpression significantly increased IL‐16 expression and secretion, and an opposite effect was observed with RBPJL (p.P476S) overexpression. Further studies also revealed that the CM of RBPJL‐overexpressing cells as well as rIL‐16 dose‐dependently enhanced T‐cell chemotaxis, proliferation and Th2/Th1 differentiation. Blocking IL‐16 with a neutralising antibody significantly attenuated these effects. Our results suggest that RBPJL might act upstream of NF‐κB to regulate IL‐16 expression and secretion, which plays a key role in the chemotaxis and proliferation of T cells, and Th1/Th2 differentiation induced by CM from RBPJL‐overexpressing cells. We also examined the expression of RBPJL and IL‐16 in primary tumors as well as liver and lung metastases. Higher expression of RBPJL and IL‐16 was found in primary and metastatic lung lesions than in liver metastases, suggesting that RBPJL knockdown and RBPJL (p.P476S) have a similar effect on T‐cell function.

The major limitation of the present study was that the p.P476S mutation of RBPJL was derived from one patient with ESCC. More ESCC patient samples would be required to identify the value of RBPJL (p.P476S) as a potential new predictor for evaluating the efficacy of anti‐PD‐1 therapy, in order to explore the therapeutic values of PD‐1 inhibitors.

**Figure 7:**
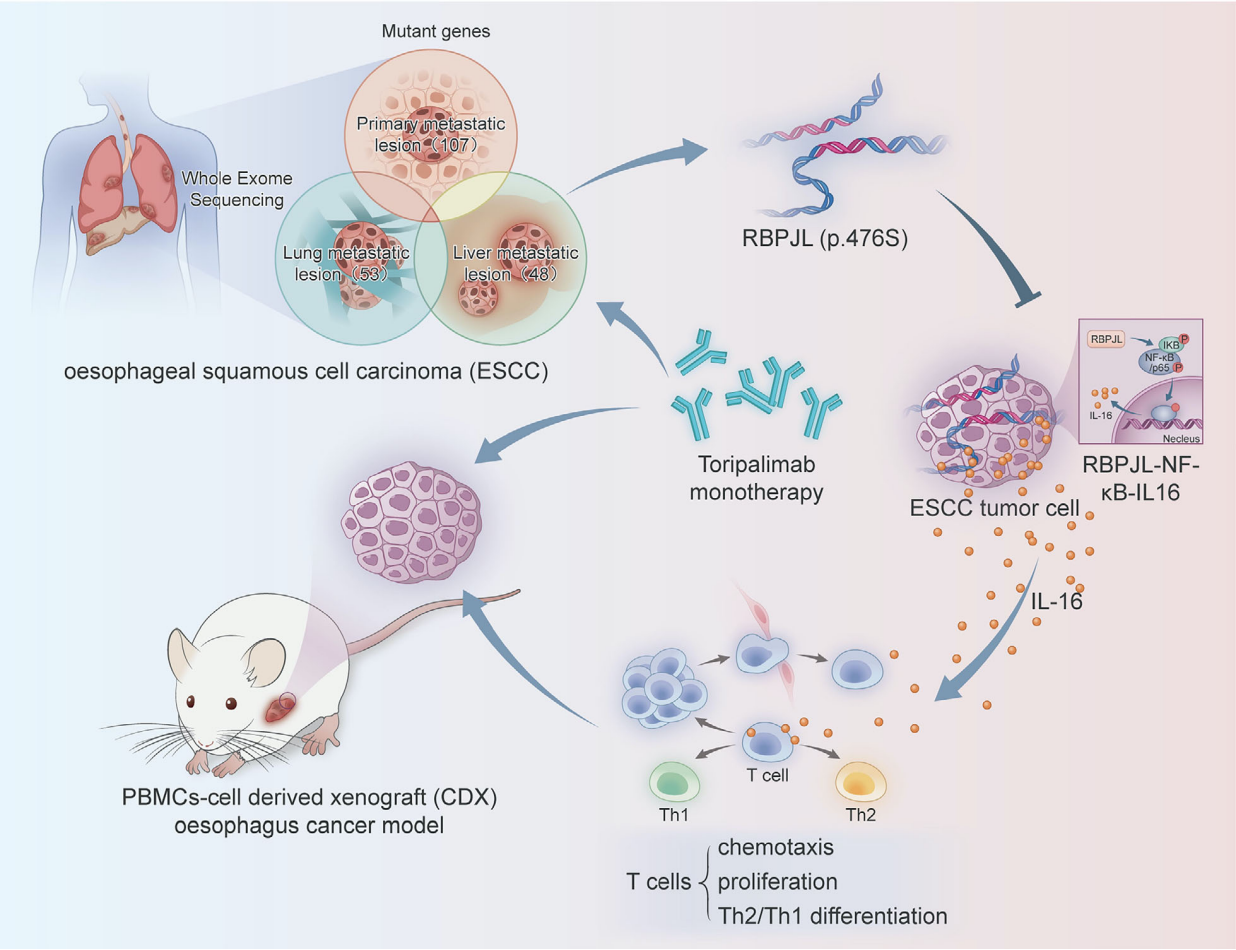
Schematic representation of the inhibition effect of RBPJL (p.P476S) on anti‐PD‐1 therapy. RBPJL (p.P476S) dampened the response of ESCC to anti‐PD‐1 treatment by reducing the chemotaxis and proliferation of T cells as well as Th2/Th1 differentiation through the inhibition of the RBPJL‐NF‐κB‐IL‐16 axis.

Based on these clinical and translational findings, we reported for the first time that the p.P476S mutation of RBPJL promotes tumor growth in ESCC and inhibits the efficacy of anti‐PD‐1 therapy in ESCC patients. Mechanically, the p.P476S mutation of RBPJL in liver metastatic lesions markedly inhibited the NF‐κB pathway activated by RBPJL, thus dampening IL‐16 production in ESCC cell lines, leading to reduced chemotaxis and proliferation of T cell and Th2 to Th1 differentiation (Figure 7). However, the detailed mechanism of action of RBPJL (p.P476S) remains to be elucidated. Taken together, our findings indicate a potential prognostic predictor for the efficacy of PD1 inhibitors in ESCC.

## Methods

### Treatment, response assessment and tumor samples

The study patient was diagnosed with stage IV moderately differentiated ESCC, located in the lower oesophagus. The patient was enrolled in a phase I clinical trial (NCT02857166) at Sun Yat‐sen University Cancer Center after failing two cycles of platinum‐ and paclitaxel‐based first‐line chemotherapy as well as seven cycles of irinotecan‐based second‐line chemotherapy. While the patient was receiving six cycles of toripalimab monotherapy (1 mg kg^−1^, Q2W; Junshi Biosciences Inc., Shanghai, China), we observed the shrinkage of the primary oesophageal lesion and the metastatic lesions in the lung, and the progression of the liver metastatic lesions. The patient was selected for analysis because of his special response pattern, availability of adequate tumor biopsy samples and clinical follow‐up data. Tumor response was assessed with computed tomography scans every 6 weeks. Pretreatment biopsies of primary oesophageal lesions and post‐treatment biopsies of primary oesophageal lesions, metastatic liver and lung lesions were conducted.

### IHC staining

The study was approved by the Institutional Ethics Committee of Sun Yat‐sen University Cancer Center (SYSUCC). IHC staining was performed as previously described.^51^, ^52^ The slides from pretreatment and post‐treatment biopsies were de‐waxed and rehydrated. The antigens were retrieved, and the slides were permeabilised and blocked before hybridisation with mouse anti‐CD4 antibody (Cat# ZM‐0418; ZSGB‐Bio, Beijing, China), rabbit anti‐CD8 antibody (Cat# ZA‐0508; ZSGB‐Bio), mouse anti‐CD68 antibody (Cat# ab201340; Abcam, Cambridge, MA, USA), mouse anti‐PD‐L1/CD274 antibody (Cat# 66248‐1‐Ig; ProteinTech, Chicago, IL, USA), rabbit anti‐RBPJL antibody (Cat# LS‐C162508; LS Bio, Seattle, WA, USA), rabbit anti‐IL‐16 antibody (Cat# DF6600; Affinity Biosciences, Cincinnati, OH, USA), mouse anti‐Ki‐67 antibody (Cat# 9449; Cell Signaling Technology, Danvers, MA, USA), rabbit anti‐CD4 antibody (Cat# 25229; Cell Signaling Technology) and rabbit anti‐CD8 antibody (Cat# 98941; Cell Signaling Technology) at 4°C overnight, followed by incubation with biotinylated goat anti‐rabbit/mouse immunoglobulin (GK500705, Dako, Glostrup, Denmark) at 37°C for 30 min. The sections were visualised using diaminobenzidine reagents. Five representative fields were identified and analysed from each section. The average positive ratio from each section was defined by the symbol and colour of stromal mono‐nucleated cells. The slides were scored independently by two experienced pathologists.

### Cell lines and cell culture

Of the ESCC cell lines used, EC9706 was obtained from TOKU‐E Company, Bellingham, WA, USA; Eca‐109 was obtained from Cell Bank, Shanghai Institutes for Biological Sciences, Chinese Academy of Sciences; and KYSE150, KYSE510, KYSE410, KYSE30, KYSE520, KYSE180, TE‐1, CE‐81T and ECa109 were all obtained from Leibniz Institute DSMZ. The cells were thawed, maintained in RPMI1640 medium supplemented with 10% FBS and 1% penicillin/streptomycin, and then cultured at 37 °C in a humidified atmosphere containing 5% CO_2_. Based on short tandem repeat profiling, no cells used in this study were found in the database of commonly misidentified cell lines, and all the cells were tested and found to be free of mycoplasma contamination by the vendors.

### Whole‐exome sequencing and identification of somatic mutations

Total DNA was extracted from the primary and metastatic tumor biopsies and subjected to whole‐exome sequencing with Illumina HiSeq 2000. For data processing, high‐quality paired‐end reads were identified for gapped alignment to the UCSC human reference genome (hg19) using BWA‐MEM. Picard (v1.84; http://broadinstitute.github.io/picard/) was used to sort and mark duplicate reads from PCR. Next, local realignment and base quality score recalibration of the BWA‐aligned reads were conducted using the Genome Analysis Toolkit (GATK4, http://www.broadinstitute.org/gatk). Somatic indels were detected by MutTect2 (v1.1.4) using the default parameter. According to standard procedures, all SNVs and indels were annotated using ANNOVAR^53^ and then filtered out with a frequency > 0.01 in the following databases: 1000 Genome Project April 2015 release and the Exome Aggregation Consortium (ExAC) database release 0.3.

### Public database analysis

The mRNA expression levels of 48 genes that had specific nonsynonymous mutations in the liver metastatic lesions at the top 15% of good prognosis and the bottom 15% of the poor prognosis of ESCC were analysed. The correlation and prognostic value were obtained from the Gene Expression Profiling Interactive Analysis (GEPIA) portal.

### Plasmid constructs

Expression vectors including full‐length and mutant cDNAs of *DTX2*, *RBPJL*, *CLEC14A* and *GABPB1* were provided and synthetised by OBiO Technology Inc. (Shanghai, China). A control transfected with EV was used in the study. The shRNAs targeting human *RBPJL* were as follows: #1, GCACCAACTTCCACCTCTT; #2, ACAGGGCTCTGCTTAACGA; and #3, GCTCAAAGGTCTCCCTCTT. All the resulting constructs were verified by sequencing, and their knockdown efficiency and specificity were validated by RT‐PCR. Cell transfection was performed using Lipofectamine™ 3000 Transfection Reagent (Invitrogen, Carlsbad, CA, USA) for shRNAs and RNAiMAX Transfection Reagent (Invitrogen) for siRNAs according to the manufacturer’s instructions.

### Isolation and preparation of T cells

Healthy peripheral venous blood was obtained from Guangzhou Blood Centre, and the study was approved by the Institutional Ethics Committee. Peripheral blood mononuclear cells (PBMCs) were isolated by Ficoll‐Paque Plus (GE Healthcare, Pittsburgh, PA, USA) gradient centrifugation, and cell viability was determined by trypan blue exclusion. Naïve T cells were purified from PBMCs by a negative selection process using a Pan T Cell Isolation Kit (Miltenyi Biotec, Bergisch Gladbach, Germany), and CD4^+^ or CD8^+^ T cells were further purified by a positive‐selection process using CD4 or CD8 T microbeads (Miltenyi Biotec).

### Chemotaxis assay

Sorted naïve CD4^+^ or CD8^+^ T cells were seeded (10^6^ per well) in the top chambers of a 6‐well transwell plate (Corning, NY, USA). The indicated CMs were added to the bottom chambers (inserts with 0.4 mm pore size). After incubation for 6 h, the chemotaxis of CD4^+^ and CD8^+^ T cells was analysed by counting the number of cells that had migrated to the bottom chamber.

### CFSE proliferation assay

Anti‐CD3/CD28‐activated purified CD3^+^ T cells were stained with a CFSE Cell Division Tracking Kit (BioLegend, San Diego, CA, USA), and they were exposed to different groups of CMs for 5 days. On day 5, the cells were harvested, and the percentage of proliferating cells was assessed by CFSE fluorescent staining and analysed by flow cytometry.

### MTS proliferation assay

Cell viability was assessed using an MTS Assay Kit (Cat# G3580; Promega, Beijing, China) by measuring absorbance at 492 nm in a 96‐well plate. The cell viability index was calculated as the absorption in the indicated treatment groups.

### T‐cell differentiation assay

Purified naïve CD4^+^ T cells were cultured in serum‐free ImmunoCult‐XF T Cell Exp Medium (StemCell, Vancouver, BC, Canada), stimulated by ImmunoCult™ Human CD3/CD28 T Cell Activator (Squirrel‐Bio, Guangzhou, China) and human IL‐2 (BioLegend), and incubated in the presence of CMs from cells overexpressing the four wild‐type or mutant genes for 48 h. Next, they were stained with CD4‐APC (BioLegend), T‐bet‐PerCP/Cyanine5.5 (BioLegend) and GATA‐3 Alexa Fluor^®^ 488 (BioLegend) for flow cytometry analysis.

### Generation of PBMC macrophages

Monocytes were purified from PBMCs by a positive‐selection process using CD14 microbeads (Miltenyi Biotec). Monocytes were cultured at 37°C in 5% CO_2_ for 7–10 days in RPMI1640 medium containing 10% FBS and 20 ng mL^−1^ human M‐CSF (PeproTech, Rocky Hill, USA). To detect the phenotype of PBMC‐derived macrophages, they were incubated with CMs from cells overexpressing the four wild‐type or mutant genes for 48 h after differentiation. The phenotype of the macrophages was analysed by staining with CD11b‐FITC, CD163‐PE and CD86‐APC for flow cytometry analysis.

### RNA extraction and RT‐PCR

Total RNA was extracted using TRIzol reagent (Thermo Fisher, Bohemia, NY, USA) and converted to cDNA according to the manufacturer’s instructions. The expression of the target genes was normalised to that of GAPDH, and the fold change was calculated as $2^{‐\Delta \Delta C_{T}}$ comparative thresholds. Specific primer sequences are shown in Supplementary table 1.

### Enzyme‐linked immunosorbent assay (ELISA)

CD3^+^ T cells were treated as indicated, and the supernatants were collected after 48 h. Next, the level of IFN‐γ was measured by using human IFN‐γ ELISA Kit (Cat# SEKH‐0046; Solarbio, Beijing, China).

### Detection and measurement of cytokines induced by gene overexpression

The detection and quantification of cytokines induced by gene overexpression were measured by Quantibody^®^ Human Inflammation Array 3 quantitative measurement of 40 human cytokines (RayBiotech, Norcross, GA, USA) according to standard procedures. Functions and pathway annotation hits of differentially expressed genes were obtained from GO classification and KEGG databases. Cytokine arrays are listed in Supplementary table 2.

### Western blot analysis

The cells were lysed with ice‐cold RIPA buffer containing complete protease inhibitors and a phosphatase inhibitor cocktail. Protein concentrations were normalised using a BCA Protein Assay Kit (Thermo Fisher Scientific, Waltham, MA, USA). Western blot analysis was performed as previously described. Primary antibodies including anti‐β‐actin, anti‐IκBα, anti‐p‐IκBα, NF‐κB/p65 and anti‐p‐NF‐κB/p65 (all from Cell Signaling Technology) were diluted to a 1:1000 ratio for each blot. All bands were visualised by enhanced chemiluminescence and quantitated by Image J analysis software.

### *In vivo* study

To imitate human tumor and human immune system interactions *in vivo*, the PBMC‐CDX (PBMCs cell‐derived xenograft) mouse model was systematically generated as follows.^54^ Briefly, EV‐, RBPJL‐ and RBPJL (p.P476S)‐treated KYSE150 (5 × 10^5^/mice in each group) were subcutaneously injected into the hind flanks of 6‐ to 8‐week‐old NSG mice. The mice were sub‐lethally irradiated (0.5 Gy) when palpable, followed by intravenous transplantation of 5 × 10^6^ human PBMCs after 4 h. Toripalimab or control IgG was administered simultaneously at a dosage of 10 mg kg^−1^ every 3 days for a total of five times. Tumor growth was monitored and calculated every 3 days from calliper measurements. The formula was as follows: volume = width^2^ × length/2. At the end of treatment, the mice were sacrificed, and peripheral blood and tumor tissues were collected for further analyses. All the animal experiments were approved by the Institutional Animal Care and Use Committee of SYSUCC.

### Statistical analysis

All the Western blots shown are representative results from at least two independent biological replicates. All bar graphs represent the means ± standard deviation. Statistical calculations were derived from at least three independent experiments and analysed by the Student’s *t*‐test (unpaired, two‐tailed) using GraphPad Prism 5.0 software, San Diego, CA, USA. A *P‐*value < 0.05 indicated statistical significance. *P < 0.05, **P < 0.01, ***P < 0.001.

## Conflict of interest

The authors declare no conflict of interest.

## Author contributions

**Lei Miao:** Conceptualization; Formal analysis; Funding acquisition; Methodology; Writing‐original draft. **Xiao‐Li Wei:** Conceptualization; Formal analysis; Project administration; Writing‐original draft. **Qi Zhao:** Data curation; Formal analysis; Software; Writing‐original draft. **JingJing Qi:** Formal analysis; Writing‐review & editing. **Chao Ren:** Investigation; Project administration. **Qi‐Nian Wu:** Formal analysis; Validation. **Da‐Liang Wei:** Methodology. **Jia Liu:** Data curation; Validation. **Feng‐Hua Wang:** Conceptualization; Investigation; Resources; Supervision. **Rui‐Hua Xu:** Conceptualization; Resources; Supervision; Writing‐review & editing.

## Ethic statement

This clinical trial (NCT02857166) was approved by the institutional review board of Sun Yat‐sen University, and signed informed consent including the collection and usage of pathological tissues was provided by the patient. This study was conducted in accordance with the ethical standards of the World Medical Association Declaration of Helsinki.

## Supporting information

Supplementary figures 1–6

Supplementary tables 1–3
