# Supplementary material for: p.P476S mutation of RBPJL inhibits the efficacy of anti‐PD‐1 therapy in oesophageal squamous cell carcinoma by blunting T‐cell responses
Source: Clin Transl Immunology. 2020 Sep 16;9(9):e1172. doi: 10.1002/cti2.1172 (PMC7507108; doi:10.1002/cti2.1172)
Supplement: Supplementary file 1 — Supplementary figures 1–6 [file CTI2-9-e1172-s001.docx]

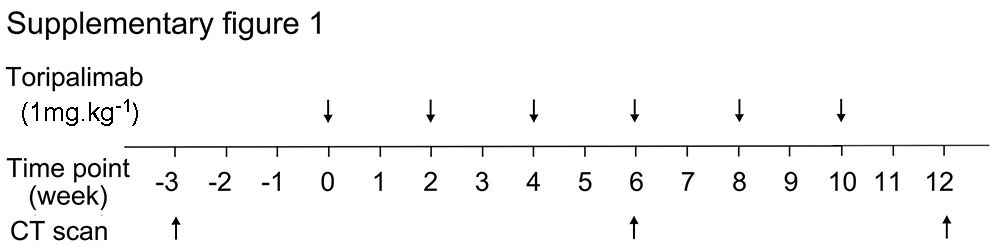


**Supplementary figure 1. Course of treatment and computed tomography scan.** One cycle of toripalimab treatment contains two infusions.


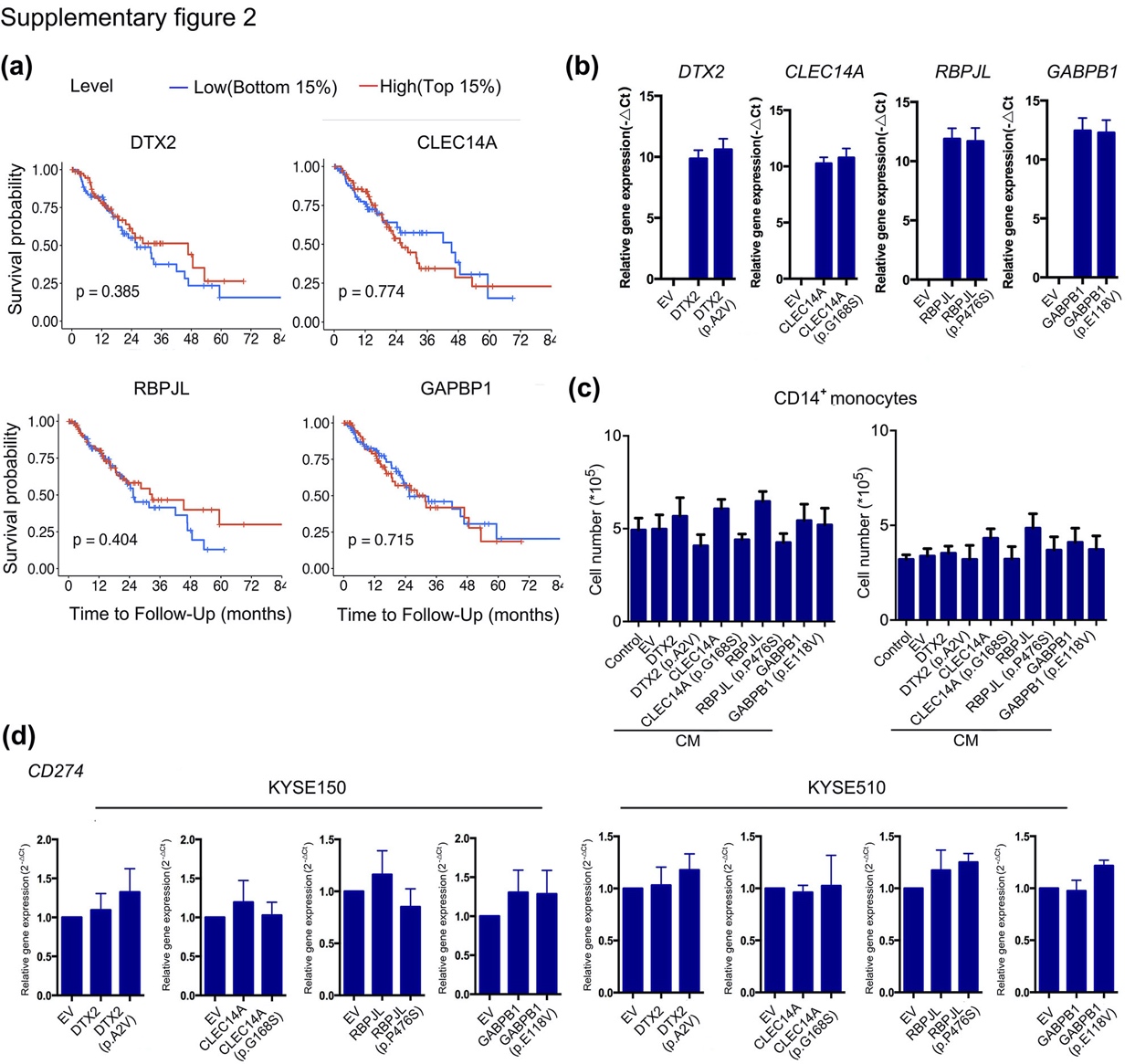


**Supplementary figure 2. CMs derived from cells overexpressing the four full-length and mutant genes could not alter the expression of PD-L1 and the chemotaxis of CD14^+^ monocytes. (a)** The mRNA expression of *DTX2*, *RBPJL*, *CLEC14A,* and *GABPB1* between the top 15 % with good prognosis and bottom 15 % with poor prognosis of ESCC was analyzed independently, and the correlation with prognosis was calculated and obtained from the GEPIA portal. **(b)** Full-length and mutant cDNA of *DTX2*, *RBPJL*, *CLEC14A*, and *GABPB1* were generated. The expression of *DTX2*, *RBPJL*, *CLEC14A,* and *GABPB1* after transfection in KYSE150 cell line was analyzed by reverse transcription-polymerase chain reaction analysis. **(c)** The chemotaxis of CD14^+^ monocytes was analyzed by transwell assay. CMs from KYSE150 and KYSE510 cells overexpressing full-length or mutant genes were placed in the bottom, CD14^+^ monocytes were seeded in the top chambers of the transwell inserts, and the number of cells migrating to the bottom chamber was counted and analyzed after incubation for 6 h. **(d)** Reverse transcription-polymerase chain reaction analysis of *CD274* (PD-L1) expression levels in KYSE150 and KYSE510 cells treated as indicated. Data in b–d are presented as mean ± standard deviation (n = 3). The data represent three independent technical replicates.


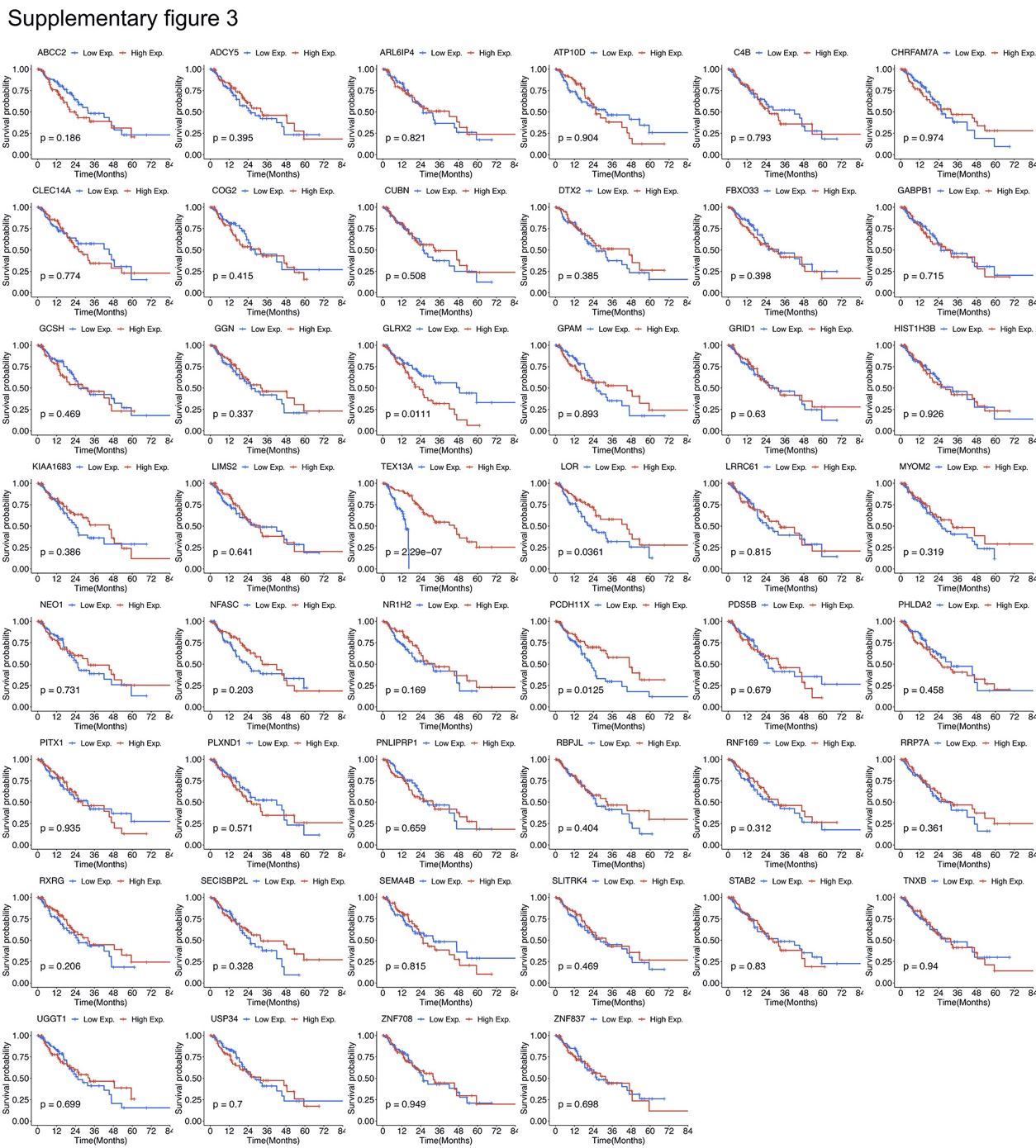


**Supplementary figure 3. The mRNA expression of 48 genes that had specific nonsynonymous mutations in the liver metastatic lesions between the top 15 % with good prognosis and bottom 15 % with poor prognosis of ESCC.** The correlation with prognosis was calculated and obtained from the GEPIA portal


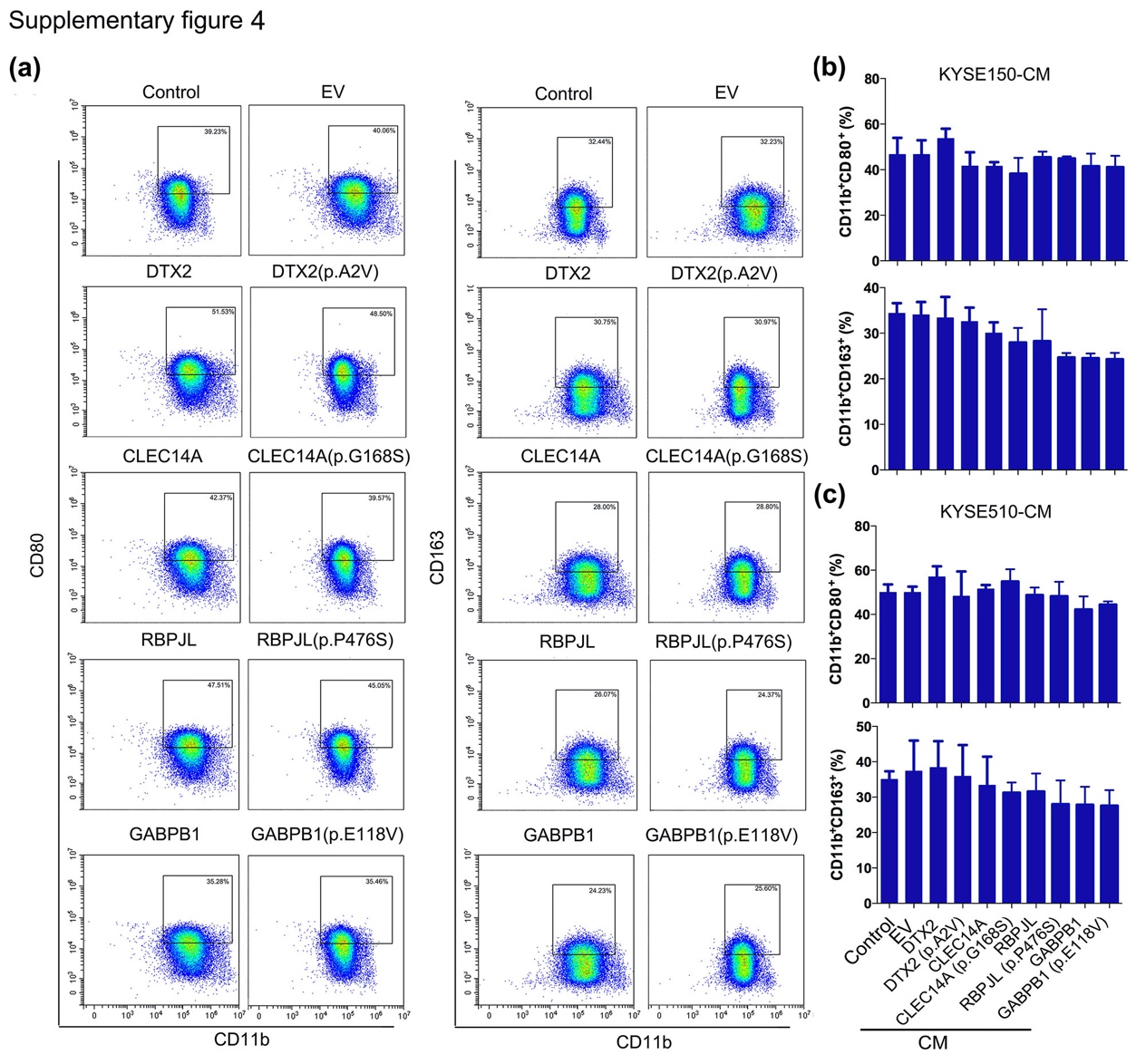


**Supplementary figure 4. CMs from the four full-length and mutant genes overexpression did not alter the phenotype of PBMC-derived macrophages.** Human PBMCs were acquired and stimulated by human M-CSF for 7 days. Flow cytometric analysis of pro-inflammatory (CD11b^+^CD80^+^) and anti-inflammatory macrophages (CD11b^+^CD163^+^) treated as indicated for 48 h. Data are presented as mean ± standard deviation (n = 3). The data represent three independent technical replicates.


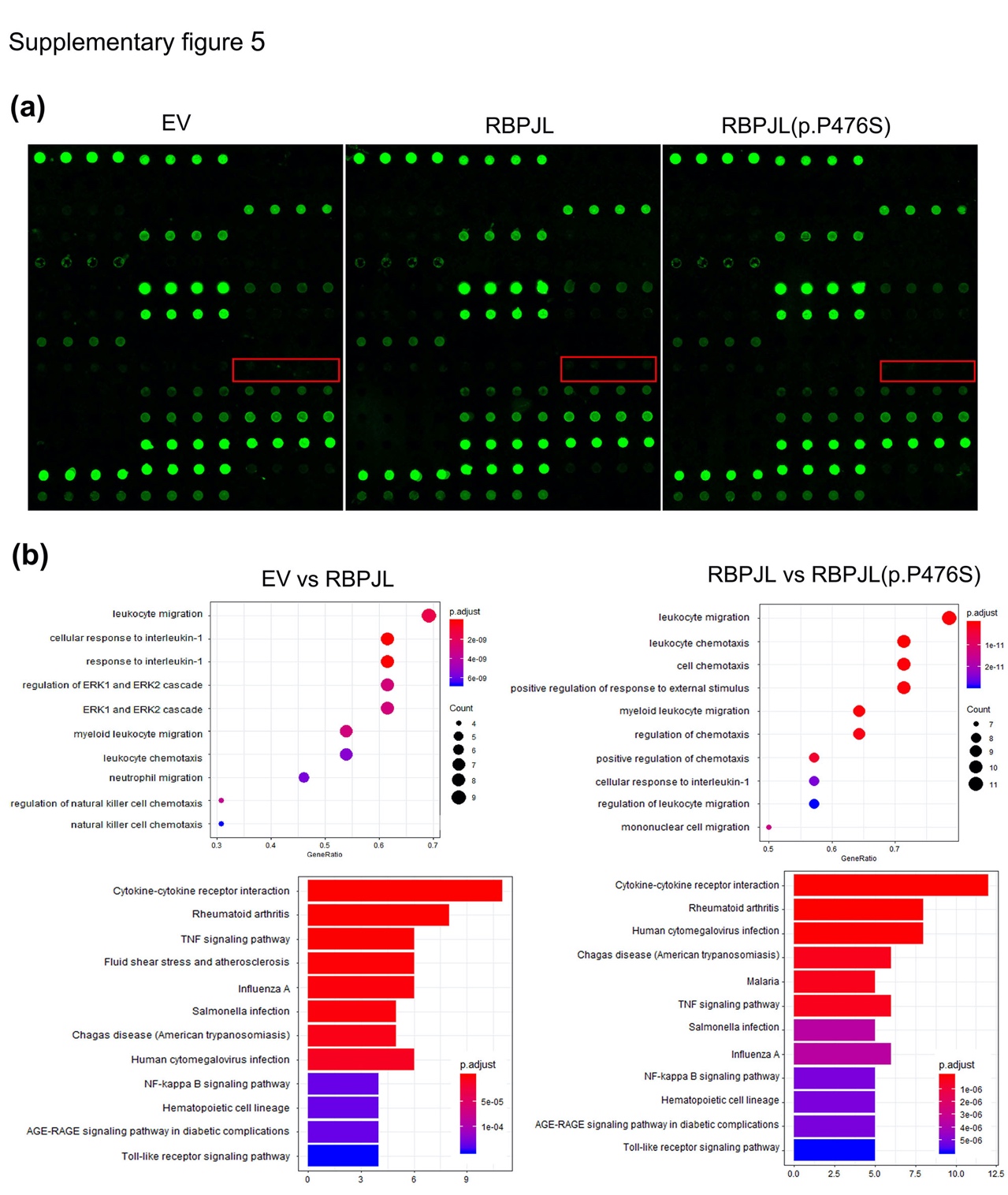


**Supplementary figure 5. Cytokine detection in EV, RBPJL, and RBPJL (p.P476S) overexpression-derived CMs. (a)** Cytokines secreted by EV, RBPJL, and mutant overexpression-treated KYSE150 were measured by quantitative measurement of 40 human cytokine arrays. **(b)** Differentially expressed gene functions and pathway annotation hits were from the GO classification and KEGG databases. The data represent three independent technical replicates.


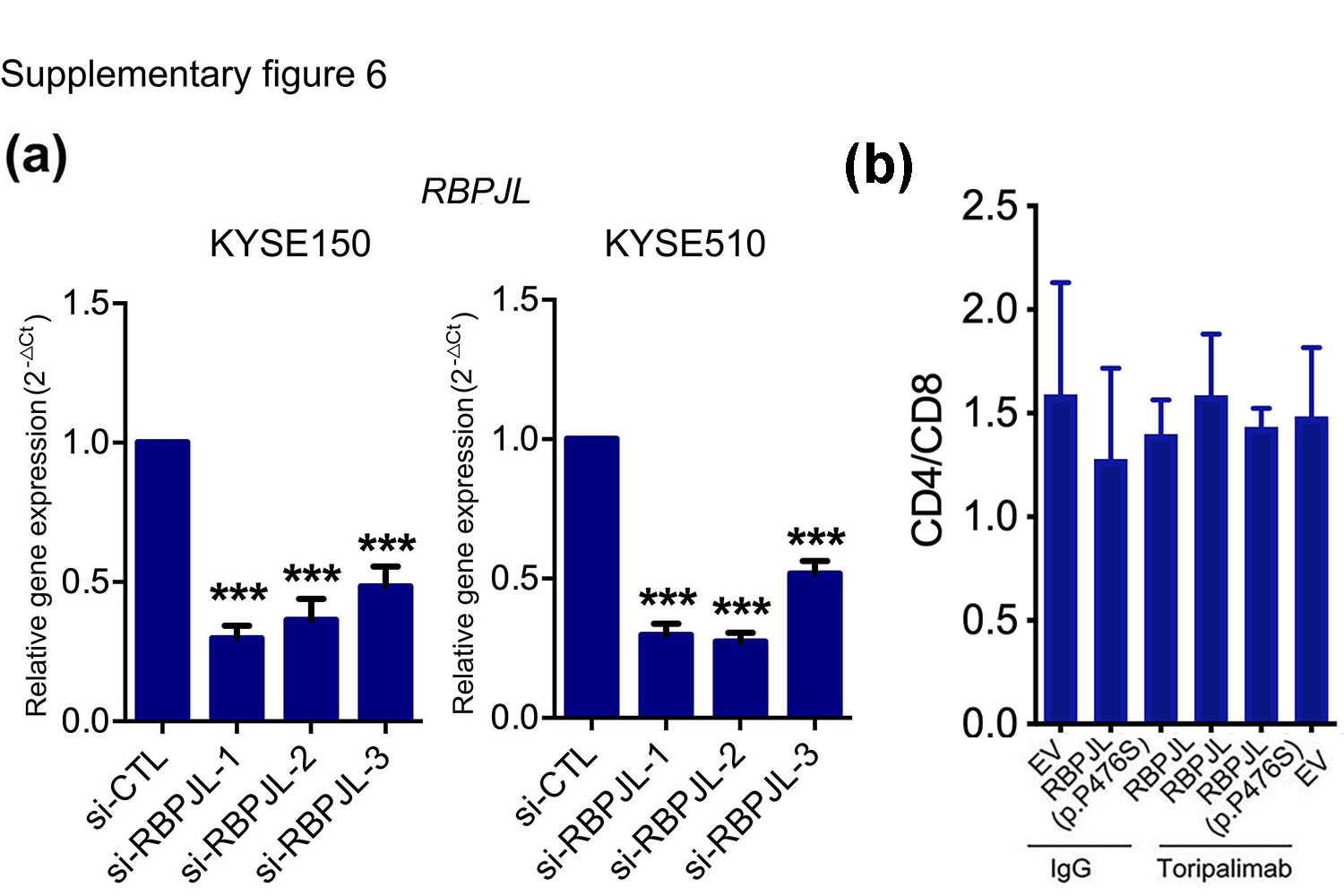


**Supplementary figure 6. (a)** RBPJL expression in KYSE150 and KYSE510 cells after transfection with siRNAs targeting human RBPJL. **(b)** The ratio of CD4/CD8 from each group in figure 6 was identified (n=6). The data above represent three independent technical replicates.
