# Supplementary material for: p.P476S mutation of RBPJL inhibits the efficacy of anti‐PD‐1 therapy in oesophageal squamous cell carcinoma by blunting T‐cell responses
Source: Clin Transl Immunology. 2020 Sep 16;9(9):e1172. doi: 10.1002/cti2.1172 (PMC7507108; doi:10.1002/cti2.1172)

**Supplementary table 1:** Sequence of primers used in Real-Time PCR


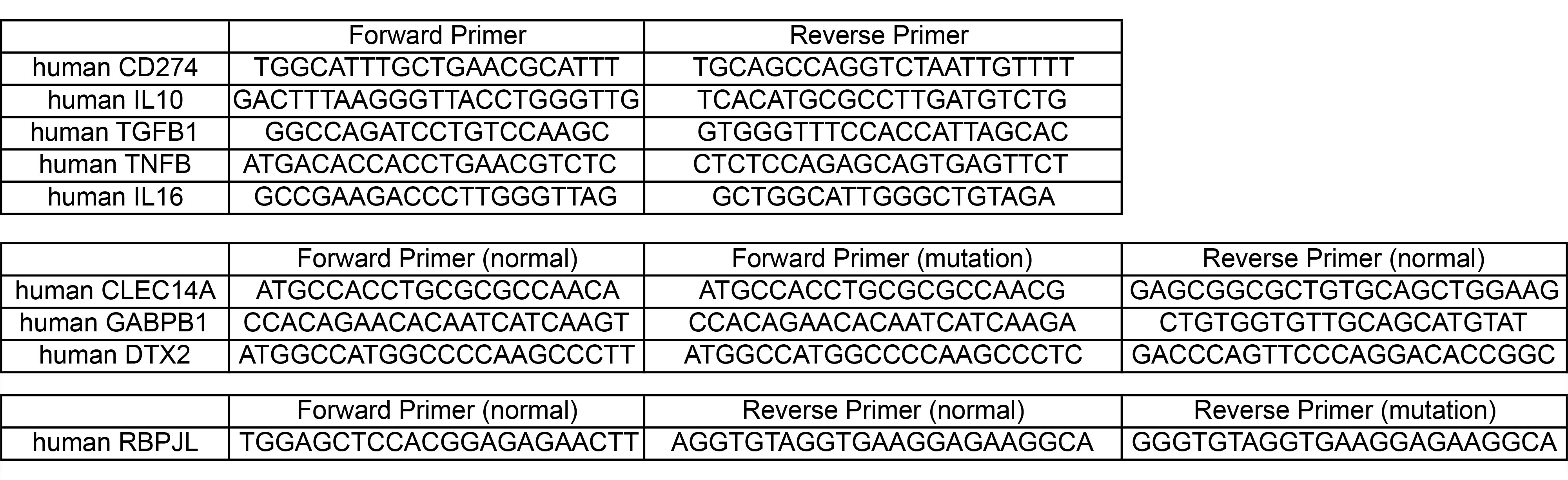


**Supplementary table 2:** Array Map of 40 human cytokines


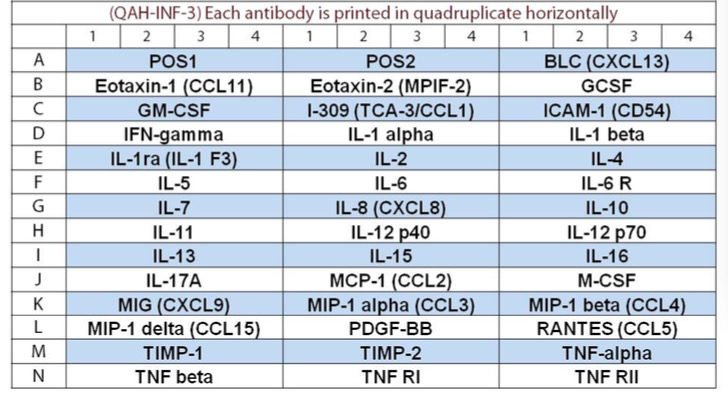


**Supplemenal table 3：**40 human cytokines comparisons after treatment


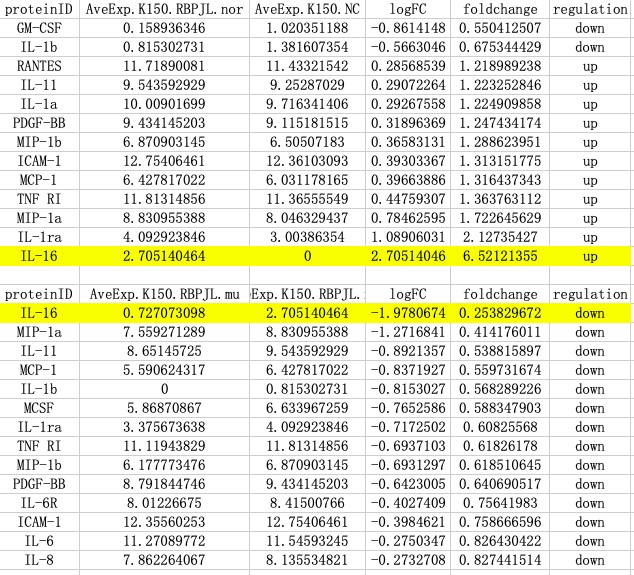

Supplement: Supplementary file 2 — Supplementary tables 1–3 [file CTI2-9-e1172-s002.docx]
